# Achieving success in clinically based research: the importance of mentoring

**DOI:** 10.1002/jmrs.234

**Published:** 2017-06-26

**Authors:** Elizabeth C. Ward, Catriona Hargrave, Elizabeth Brown, Georgia Halkett, Peter Hogg

**Affiliations:** ^1^ Centre for Functioning and Health Research Metro South Health Brisbane Queensland Australia; ^2^ School of Health & Rehabilitation Sciences The University of Queensland Brisbane Queensland Australia; ^3^ Cancer Services Princess Alexandra Hospital Metro South Health Brisbane Queensland Australia; ^4^ School of Clinical Sciences Queensland University of Technology Brisbane Queensland Australia; ^5^ Department of Radiation Oncology Princess Alexandra Hospital Brisbane Queensland Australia; ^6^ School of Nursing Midwifery and Paramedicine Faculty of Health Sciences Curtin University Perth Australia; ^7^ Directorate of Radiography University of Salford Greater Manchester UK

**Keywords:** Mentoring, clinical research, research capacity building

## Abstract

Within the professions of radiation therapy and medical imaging, clinician led research activity is becoming more prevalent. However, more is needed. A key component of continuing to develop professional groups who are both research active and producing high quality clinical research, is research mentoring. The authors of this paper share a common interest in enhancing research capacity through research mentoring within the health workforce, and came together to run a workshop on this issue at the 11th Annual Scientific Meeting of Medical Imaging and Radiation Therapy (ASMMIRT 2016) conference in Brisbane. Theory, clinical insights and issues regarding research mentoring were raised in the workshop as were the benefits of having dedicated research positions embedded within the health workforce to help provide support and build capacity. Key elements from this workshop are shared within this article, with the objective to encourage clinicians and clinical researchers to invest the time and effort into seeking and providing good quality research mentoring. A single service example is used to demonstrate how this can lead to enhanced research engagement and productivity.

## Background

To ensure clinical services continue to meet the growing and ever changing needs of modern healthcare, greater importance is being placed on patient‐focused research that is driven by the clinical workforce.^1^, ^2^, ^3^, ^4^ Across allied health professional groups, including medical radiation science professionals there is strong support for this initiative. However, it is acknowledged that engaging the clinical workforce in research has its challenges. Challenges that have been reported within radiation therapy include limited time, research facilities, access to mentors and workforce capacity.^4^, ^5^, ^6^

Developing, supporting and growing a clinical workforce that is research aware, engaged and appropriately skilled to conduct clinical research requires diverse, but inter‐related strategies.^3^ Arguably a key strategy is ensuring clinicians have access to research mentors.^7^, ^8^, ^9^ Mentoring is more than just supervision, and achieving effective mentoring requires numerous factors, and can be enhanced by mentor training.^8^, ^9^, ^10^ Mentoring busy clinicians to develop research skills and capacity requires a unique understanding of clinical pressures and workforce limitations. By working together, the mentor and mentee can ensure that any research planned is appropriate for the setting, and that both personal and departmental requirements have been considered. This helps to provide a realistic scenario for various stages of research, from conception through data collection to dissemination, and helps to engage clinicians through research questions that target important clinical issues. The mentors then have a key role in supporting, advocating and facilitating the path for these developing researchers as they grow and develop skills, ultimately supporting the management of complex projects that improve practice.

Although research mentorship is important for individuals at all stages of their research career; our article focuses on issues of mentorship for novice/early researchers which were discussed at Annual Scientific Meeting of Medical Imaging and Radiation Therapy (ASMMIRT) 2016 conference. The 2 h seminar was attended by 25 participants of which one‐third were medical imaging technologists and two‐thirds radiation therapists. Three‐quarters of the audience were clinicians, with the rest holding academic positions. Attendees came from multiple states in Australia with one international participant. The workshop covered theory and personal insights on mentoring and a final discussion session with participants. Discussions during and after the workshop with participants identified elements they found most beneficial, and informed the selection of content shared in this commentary paper.

## How the Mentor Can Help

When developing a new ability, such as research, it is helpful to have an experienced person give advice, support and mentorship.^6^, ^7^ Their involvement can speed up the learning process, identify more efficient ways of doing things and give insight into what abilities are needed and at what level. They can also help with reflection, to determine whether adequate performance is being achieved and whether corrective action is necessary. Mentorship from experienced people can make the seemingly impossible, possible.

Mentorship helps the mentee identify strengths and areas for development, that may be individually focused (e.g. a skill) or environmental. Environments conducive to research are those that allow research and researchers to flourish. Cultural and resource factors are at play and often these are hard to influence by the novice researcher. However, these can be acknowledged and accounted for in the mentoring process.

## Mentoring Individual Novice and Mid‐Career Researchers: Reflections on Two Case Studies

The following cases provide examples of how an experienced mentor can help early career researchers overcome barriers and achieve success in research.

‘John’, a mid‐career diagnostic radiographer, wanted to develop a research career. He holds multiple degrees and is ambitious and willing to learn. He identified and established a relationship with a mentor based in another country, and together they quickly realised John's environment was extremely busy with no opportunity for research, or capacity to develop a research network. Through regular Skype/WhatsApp/email exchanges, the mentor helped John examine these challenges and a way forward was planned. Through networking and support from the mentor, John is now part of a multi‐professional international research team and publishes 1–2 articles annually. Within the next year, several of his journal articles will be submitted in a thesis (PhD by published works). He benefits from the international research team as it helps him identify his learning needs and achieve research outputs. He is now considering future roles and opportunities that could provide enhanced opportunities to continue to develop his research career.

‘Jenny’, an experienced consultant [breast] radiographer, works in a demanding clinical role. She holds two professionals qualifications and a Master of Science degree. For consultant radiographers research is a role expectation. However Jenny, like many others was having difficulty achieving this. Recognising her own personal desire and professional need to engage in research, she identified a local university‐based mentor, who provided assistance and support via regular face‐to‐face meetings. Although extremely busy, Jenny works in a supportive environment and is allowed half a day per week for research, consistent with the terms of her contract as a consultant radiographer. Hence, expectations with the mentor were set against this, and Jenny initiated a level of engagement with research that was balanced against the demanding aspects of her work. With help from the mentor, a new research area was created with Jenny in mind and a research team was established. Through mentorship, Jenny was encouraged to also engage in key facilitation roles in other projects, which fitted well with her clinical role expectations. She achieved success, and has maintained research outputs of 1–2 publications per annum over the past 10 years. This consistent level of journal output has been achievable over time through maintaining collaborations as a co‐author with various university research teams. While Jenny has been a co‐author on publications to date, she is now keen to have an opportunity to lead a project and hold a key author position (e.g. first, second or last). Consequently, recent discussions with her mentor now seek to extend her opportunities to take on a research leadership role.

## Getting the Most Out of the Mentor‐Mentee Relationship

Successful clinical researchers can cite many ways their mentors help to build their personal capacity and career. For some, initially finding their mentor may have occurred as part of a structured mentoring program. For others, finding a mentor was part of a personal journey, in which they reflected on current needs and independently found an appropriate mentor. For many, their mentor is typically a more senior researcher in their professional field; however, mentors may also come from leaders in related/cognate fields, who often can bring different perspectives and new ideas to old problems. Regardless of how the relationship is established, it is acknowledged that both the mentor and mentee need to contribute to the relationship to ensure it is beneficial to both.

When the relationship is being established, the mentor and mentee need to consider their roles in the relationship and what to expect from each other. The mentor's role will consist of providing individual attention, expertise, support and encouragement, and open and honest feedback. It is also part of the mentor's role to conduct their own self‐reflection, to ensure they are giving their best. The mentor needs to be able to support the mentee throughout the highs and lows, providing support and inspiration particularly at difficult points in certain projects (e.g. developing the design, grant writing, dissemination and write‐up) or stages of the mentees career (e.g. promotion). In comparison, the role of the mentee includes establishing clear goals and expectations, an eagerness and open approach to learning, willingness to ask for help, willingness to take risks, and trusting their mentor.^11^ The mentee also needs to be able to accept constructive feedback, corrections and even failures, and take a proactive role in managing their research project/research career. Some key strategies for sustaining a mentor‐mentees relationship are outlined in Table 1.^8^, ^9^, ^11^, ^12^, ^13^

**Table 1:** Skills beneficial to developing and sustaining a mentor‐mentee relationship.^8^, ^9^, ^11^, ^12^, ^13^

Skill	Strategy
Communication	Discussion about roles, expectations and feedback (remember all mentees are different) Set boundaries Hold regular meetings Meeting summaries – mentee's role, mentor needs to confirm what has been discussed Checking back with each other on agreed plans and progress
Being there	Act as a sounding board and listen
Give direct feedback	Constructive feedback that is helpful Offer advice if mentee asks for it
Set clear goals	Think big picture and small picture. Big picture: Conducting a research project Gaining research funding Writing a manuscript Networking Career development Support and encouragement Making independent researcher Integrating research into clinical practice Small picture: day to day running of project
Evaluate people's strengths, needs and aspirations	Find out what the mentee needs Find out how the mentor can help Continually discuss what is needed from each other
Share experiences	Discuss previous research projects Talk about previous success Talk about when things have gone wrong Discuss ways of negotiating the clinical setting depending on previous experiences
Make time	In the clinical setting it may be difficult to find time to work together. For example in some workplaces meetings need to be held before clinical time starts. Respect the needs and time availability of both the mentor and mentee Seeking additional mentorship from others may also be beneficial if a mentor is unavailable for a period of time
Respect confidentiality	Not everything discussed in the mentoring relationship should be discussed outside of the meeting Highlight what is confidential during meetings Both parties need to respect each other's confidentiality – you may wish to set up a confidentiality agreement
Create opportunities to learn on the job	Mentee may be able to participate in conduct of research projects that mentor is leading Mentee may be able to work with other research team members to learn skills they need Mentee can learn through practising skills and then applying them to own project
Measure progress	Regular discussion of how mentee is progressing Use as opportunity to build confidence
Provide motivation and acknowledge achievements	Celebrate small and big wins Goal setting will assist in providing motivation Make sure achievements are acknowledged as this will provide motivation
Networking and Team collaboration	Mentor can provide links to other team members and potential collaborators Research projects within the team may be similar; opportunities for collaboration and learning

Ideally, the mentoring relationship is both ongoing and evolving as exemplified in ‘Jenny's’ case above. For many people, they will establish a long‐standing relationship with a mentor that spans key stages of their research development. When strong relationships are formed, an ongoing mentorship arrangement can become highly valued by both parties.

## A Service Example: The Queensland Health, Radiation Therapy Research Mentoring experience

Organisational support is fundamental to help grow a sustainable research culture that is focused on translating findings into practice.^1^, ^14^ Recognising this, in 2007 the health practitioner workforce of Queensland Health, the public health service for residents of Queensland, Australia, negotiated the health practitioners certified agreement which incorporated a ‘research package’. The package provided funding for building workforce research capacity,^1^ supporting dedicated research positions for building research capacity within the health practitioner workforce.

From this initiative, the radiation therapy (RT) principal research fellow position was established as a state‐wide position in 2009. As a state‐wide position, the role supports four Queensland Health Radiation Oncology departments, consisting of approximately 231 FTE staff. All departments are located in tertiary hospital settings and manage a large percentage of complex cases as well as providing specialist services (e.g. paediatric radiotherapy, stereotactic and ablative radiotherapy, Gamma Knife, tomotherapy, brachytherapy). The research fellow's role is to develop research activities aligned with achieving integrated service delivery that are both patients‐centred and evidence‐based, and to build a sustainable and collaborative research culture across the services. Achieving these goals requires substantial investment by the research fellow in research mentoring and networking; for both individual researchers and teams. Strong links between the research fellow's position and other researchers within Queensland Health and the staff within the university sector are also actively sourced to help increase mentoring/leadership capacity.

Successful research mentoring of RTs across all sites within Queensland Health requires the research fellow to appreciate the clinical context and environment of each setting, as well as understanding the research experience and interests of the individuals in each service. To ensure appropriate engagement and supports are available, regular support and research mentoring for the RT workforce occurs through weekly face‐to‐face meetings for services in the local metropolitan area, while regular video‐conferences along with 1–2 site visits each year are held with departments located further away. Success of the research fellow's role to build a sustainable research culture is also heavily dependent on fostering local RTs to become research mentors. One strategy which has proven very successful is to identify clinicians in each service to become departmental RT research coordinators. These are clinicians with interest and experience in research who can serve as the ‘go‐to‐person’ within each department. For example, the Princess Alexandra Hospital‐Radiation Oncology service is one of the four clinical services supported by the research fellow position. Located in a tertiary hospital setting, it employs 68 FTE radiation therapists. When developing the Princess Alexandra Hospital‐Radiation Oncology research program, a local staff member was identified to become the RT departmental research coordinator. This person was selected based on their experience as a clinical researcher, their motivation to lead and build the program, and their commitment to educating the staff group on the value and conduct of research. The role of the position is to assist the development of projects that aligned with clinical practice, and enable staff to see the value of research to their daily clinical practice. They also provide a local, approachable mentor for staff to discuss research ideas, questions and issues experienced, develop research projects, provide guidance when needed and facilitate collaboration with other disciplines. As staff who hold these positions are often actively engaged in research themselves, they also help to inspire other staff to be involved in research, modelling ways to become ‘research engaged’ within a clinical role. The departmental RT research coordinators are also responsible for training and preparing other departmental staff (who are developing their own research skills), to take on aspects of research mentoring. This approach is proving to be an important component in expanding the mentoring support available for new researchers and building clinician engagement in research.

Reflection on the past 8 years of this mentoring/support model has shown that different levels and types of support are needed within the clinicians/teams. For example, new graduates with little to no research skills/experience, benefit more from step by step directed mentoring in research skills development as well as continuous active encouragement. This approach helps to provide a positive first clinical research experience that builds their clinical confidence and confidence to undertake future research. In contrast, for clinicians who may have been involved in quality improvement activities and have some research skills, then mentoring support/assistance is often best tailored to developing specific research skills/processes such as ethics submissions, statistics or writing the paper. Finally, for those clinicians with a track record of research experience, the research fellow/departmental coordinator's role for those clinicians tends to focus on developing and expanding the clinician's research networks and collaborations in order to provide further opportunities.

**Figure 1:**
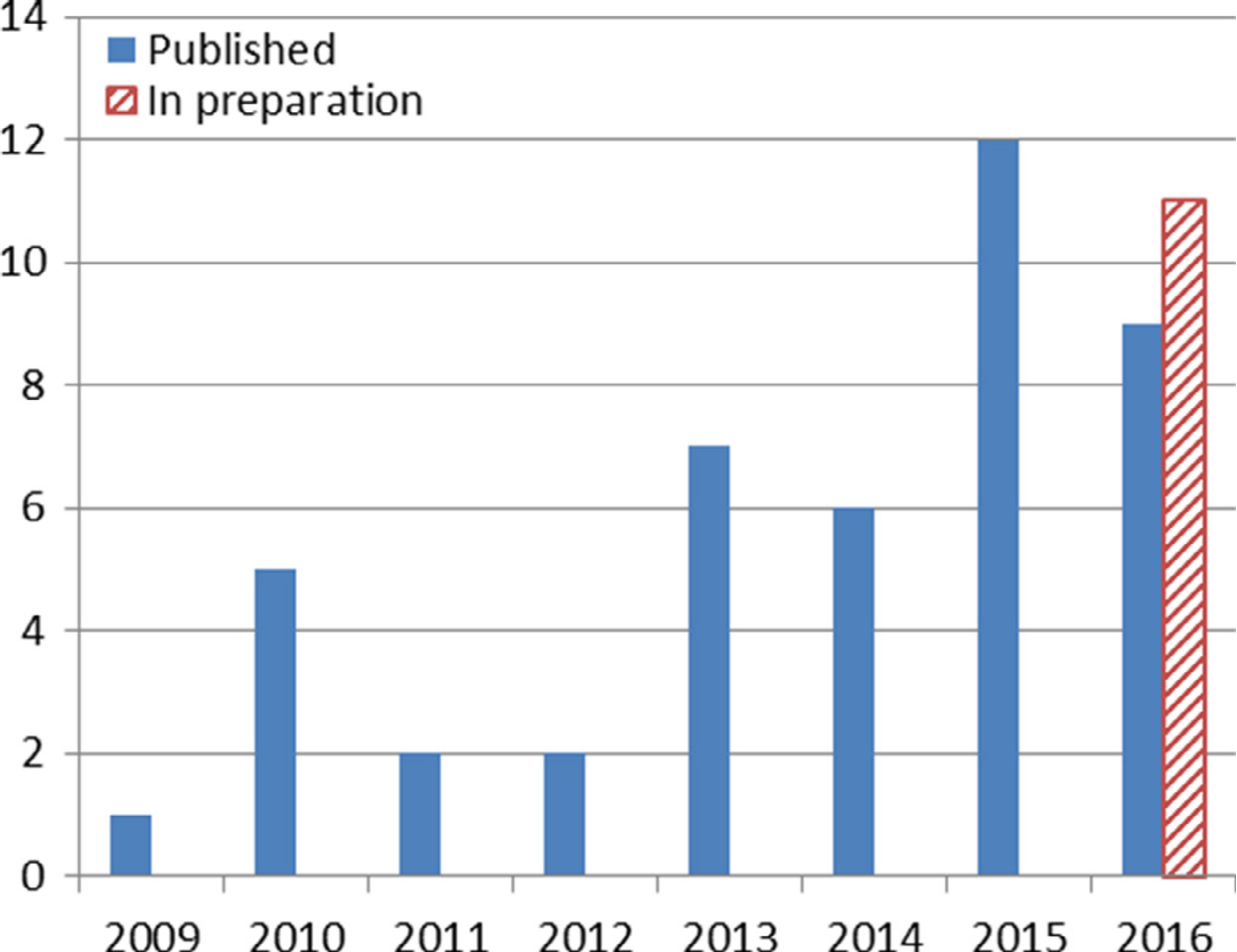
Queensland Health RT department publications 2009–2016 (position commenced in 2009).

Through the research mentoring and support provided from both the research fellow position and the local departmental RT research coordinator, RT departments within Queensland Health have been successful in increasing the numbers of RTs being involved in research projects, increasing the numbers of staff with research skills and encouraging more to take on mentoring roles to disseminating their knowledge and experience to others, supporting a steady positive growth in research outputs (Fig. 1).

## Summary

Mentoring is a critical component in building research capacity within the clinical workforce. Examples within the current paper highlight how clinicians can use mentors to help develop their clinical research skills. The case example of the research mentoring provided through the Queensland Health research fellow and local departmental research coordinator positions demonstrate how strategic gains can be achieved through active engagement in research mentoring within clinical services.
